# Industrial Biotechnology Based on Enzymes From Extreme Environments

**DOI:** 10.3389/fbioe.2022.870083

**Published:** 2022-04-05

**Authors:** Noha M. Mesbah

**Affiliations:** Faculty of Pharmacy, Suez Canal University, Ismailia, Egypt

**Keywords:** extremozyme, extreme environment, single amplified genome, metagenomics, halophile, alkaliphile, thermophile, psychrophile

## Abstract

Biocatalysis is crucial for a green, sustainable, biobased economy, and this has driven major advances in biotechnology and biocatalysis over the past 2 decades. There are numerous benefits to biocatalysis, including increased selectivity and specificity, reduced operating costs and lower toxicity, all of which result in lower environmental impact of industrial processes. Most enzymes available commercially are active and stable under a narrow range of conditions, and quickly lose activity at extremes of ion concentration, temperature, pH, pressure, and solvent concentrations. Extremophilic microorganisms thrive under extreme conditions and produce robust enzymes with higher activity and stability under unconventional circumstances. The number of extremophilic enzymes, or extremozymes, currently available are insufficient to meet growing industrial demand. This is in part due to difficulty in cultivation of extremophiles in a laboratory setting. This review will present an overview of extremozymes and their biotechnological applications. Culture-independent and genomic-based methods for study of extremozymes will be presented.

## 1 Introduction

Bioprocess technology is the use of entire living cells or their components (e.g. enzymes, metabolites) for chemical transformations (Moser 1988). Bioprocess technology has great potential for production of numerous products. Bioprocess technology is based on biocatalysis, which allows manufacture of products in bulk by utilizing enzymes, and is used in diverse sectors such as food, textile, fine chemicals, pharmaceuticals, and therapeutics (Katsimpouras and Stephanopoulos 2021). The remarkable capabilities of enzymes have revolutionized biotechnological processes. Enzymes are greener, sustainable substitutes to the use of chemicals for industrial processes. Enzyme-catalyzed reactions are specific and produce less by-products, and any by-products are of low toxicity. Enzyme immobilization and reuse is economic and environmentally feasible, and enzyme inactivation is possible (Madhavan et al., 2021). Enzymes have application in a variety of fields, including technical applications, food processing, biofuel production and organic synthesis by the pharmaceutical and cosmetics industries (Li et al., 2012). The detergent industry uses enzymes for breakdown of protein-, fat- and starch-based stains. The textile industry utilizes cellulases to soften fabric and improve color. The pulp and paper industries use amylases, cellulases, mannanases and xylanases to reduce viscosity of mixtures, improve product softness, increase brightness by degradation of residual glucomannan and efficient pulp-bleaching (Araújo et al., 2008). Lipases and lactases are used in the manufacture of cheese and production of lactose-free dairy products. Amylases and other carbohydrate hydrolases are used for improving dough handling and stability in the baking industry, and for clarification and viscosity control in the juice industry (Ribeiro et al., 2010; Li et al., 2012). Cellulases, hemicellulases, and xylanases are used for degradation of lignocellulose in production of bioethanol (Rezania et al., 2020). Most of these processes occur under harsh conditions, such as high temperature and extremes of pH, in addition to low-water activity. In the pharmaceutical industry, lipases, nitrilases, esterases, amidases, dehydrogenases, oxidases, proteases, and others, are used for chemical transformations. Many of these transformations occur under non-aqueous conditions in non-conventional media, such as organic solvents, ionic liquids and supercritical fluids (Lozano et al., 2015). The cosmetics industry incorporates proteases such as papain and subtilisin in skin care products to produce a peeling-effect, and uses peroxidases and polyphenol oxidases in hair dyes (Sanchez and Demain 2011).

From an industrial point of view, the ideal biocatalyst must be stable and active under processing conditions, have suitable substrate specificity, regio-and enantioselectivity, be efficient and produce the required product within a suitable time with little to no byproducts and minimal product-inhibition (Lorenz and Eck 2005). Most enzymes available originate from mesophilic organisms, and their narrow range of stability limits their use in many applications (Sysoev et al., 2021). Enzymes derived from extremophiles, called extremozymes, overcome these limitations.

Extremophiles are organisms which survive and thrive under conditions considered hostile from an anthropogenic point of view. Extreme environments can be natural, such as hypersaline lakes, hydrothermal vents, volcanos, arid deserts, the deep ocean, the upper atmosphere, or artificial, such as spacecrafts and operating rooms (Merino et al., 2019; Schröder et al., 2020).

**FIGURE 1:**
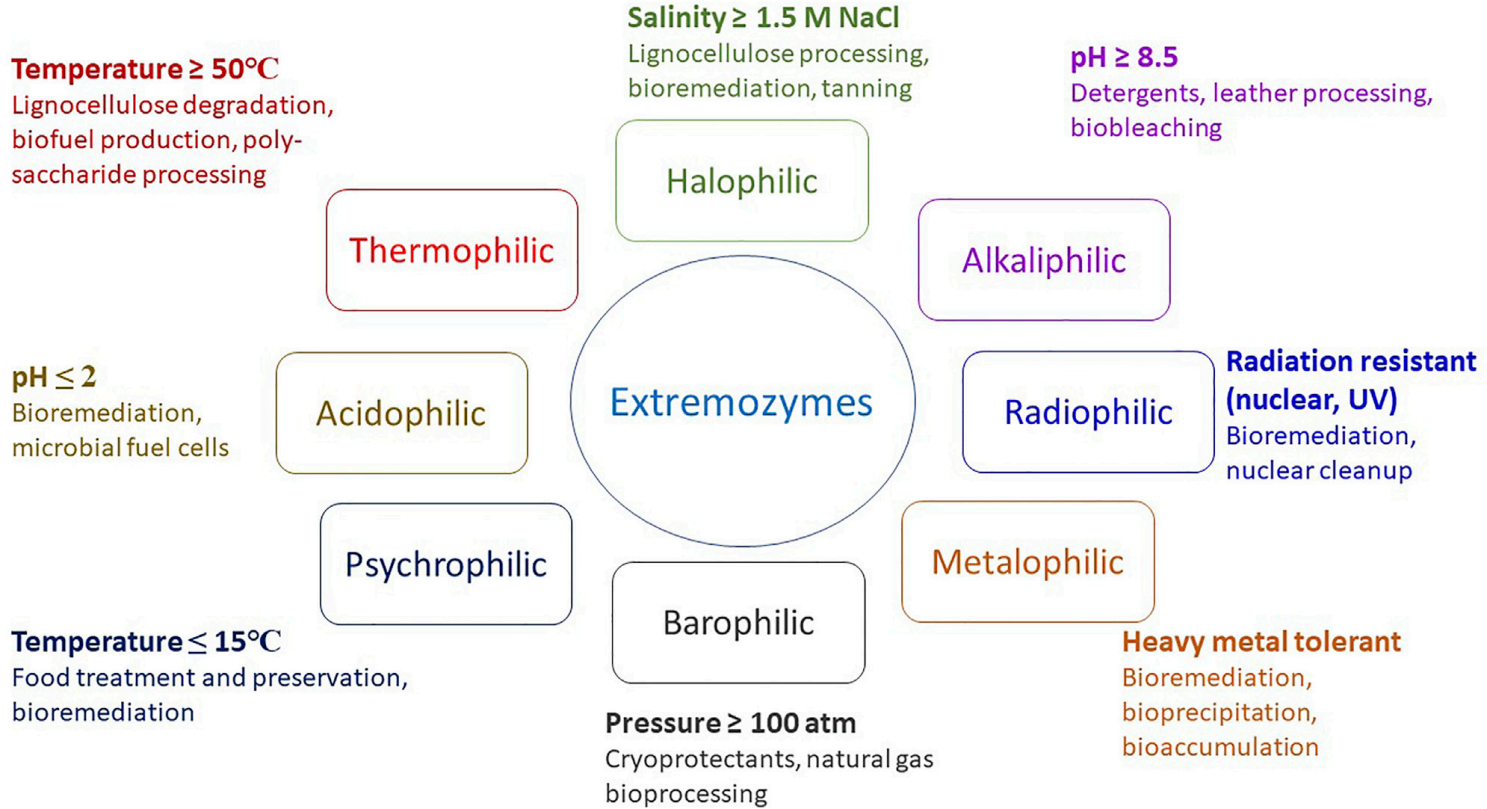
Extremozymes and their potential applications.

Extremophiles have developed diverse mechanisms and strategies to adapt to extreme conditions. The diversity of extremophiles and extreme conditions promises biocatalysts able to withstand harsh industrial conditions with higher efficiency (Elleuche et al., 2014; Jorquera et al., 2019). Extremozymes can perform reactions under a broader range of conditions compared with mesophilic enzymes. Extremozymes are classified according to the nature of their natural habitat, and include thermophilic, psychrophilic, acidophilic, alkaliphilic, halophilic, xerophilic and barophilic enzymes in addition to others (Sarmiento et al., 2015; Dumorné et al., 2017; Jin et al., 2019; Mutlu-Ingok et al., 2022). Extremozymes can be polyextremophilic, being stable and active under multiple extreme conditions such as high temperature, high salinity and alkaline pH, high salinity and low temperature, and high temperature and extremes of pH (Figure 1) (Akal et al., 2018; Laye and Dassarma 2018; Mesbah and Wiegel 2018; Mesbah 2019; Karan et al., 2020; Liu Z. et al., 2020; O’Hair et al., 2020; Wanyonyi and Mulaa 2020).

Demand for industrial enzymes is growing steadily. The global enzymes market was valued at $6.4 billion in 2021, and is projected to reach $8.7 billion in 2026 at a compound annual growth rate (CAGR) of 6.3% from 2020 to 2026 (Research B 2021). Difficulties with current industrial biotechnology are attributed to limited enzyme stability under harsh industrial processing conditions, microbial contamination, poor recyclability of biocatalysts, and limited capacity for synthetic reactions (Chen and Jiang 2018). Biosynthetic reactions using hydrolases are typically performed in the presence of solvents, where the low water activity shifts the thermodynamic equilibrium of the reaction to the synthetic direction, and suppresses aqueous side reactions (Carrea and Riva 2000). Attempts at improving properties of mesophilic enzymes through chemical and/or genetic modification and immobilization are time consuming, expensive, and not effective due to alteration in reaction rates, enzyme specificity and stability (Sysoev et al., 2021). Extremozymes are natural alternatives adapted to function under extreme conditions.

The major source for isolating and purifying extremozymes was based on cultivation of the source extremophile, followed by screening for enzyme activity and enzyme purification, either directly from the source microorganism or through heterologous expression. However, it is estimated that greater than 99% of prokaryotes cannot be cultured in laboratory settings. Uncultured microorganisms are referred to as “microbial dark matter” (Bernard et al., 2018). The past 2 decades have seen advancement of environmental genomics, where bulk DNA is isolated from an environment and screened for enzyme sequences and activity. Single amplified genomics, where individual cells are separated from an environmental sample, provides information on each cells genome and metabolic capability (Sysoev et al., 2021). Despite great advances in community genomics and single amplified genomes, to date few extremozymes have been functionally characterized using these techniques. This is partly due high sequencing costs, and lack of robust, reliable functional annotation of genomic data. This review will highlight the importance and biotechnological applications of extremozymes, and show how developments in genomics, sequencing and bioinformatics contributed to the bioprospecting of novel extremozymes.

## 2 Extremozymes

### 2.1 Thermophilic Enzymes

There are many advantages to carrying out reactions at high temperatures, including decreased risk of microbial and/or fungal contamination, increased solubility of substrates and faster hydrolysis rates. Thermophilic extremozymes have maximal activity at temperatures from 50 to 125°C (Han et al., 2019). Disulfide bridges play a major role in thermal stability as they decrease the entropy of a protein’s unfolded form, and extended disulfide bridges, particularly from the N-terminal side, led to enhanced thermostability of several enzymes (Mallick et al., 2002; Liu et al., 2016; Tang et al., 2017; Landeta et al., 2018; Han et al., 2019). Other factors such as compact (β/α)_8_ barrel folding, shorter loops and helices, salt bridges, surface charge and inner hydrophobic amino acids and interactions maintain conformation of thermophilic extremozymes (Han et al., 2019). Hyperthermostable enzymes with maximal activity above 70°C have a large number of charged amino acids, and ionic interactions play a major role in stabilization (Karshikoff et al., 2015). Thermophilic enzymes retain their thermostabilities even when cloned and expressed in mesophilic hosts, indicating that their thermal adaptions are genetically encoded (Vieille and Zeikus 2001).

Thermophilic extremozymes are the most widely used of the extremozymes. The first, large scale use of a thermophilic extremozyme was that of the DNA polymerase (*Taq* polymerase) from thermophilic *Thermus aquaticus*, isolated from hydrothermal vents in Yellowstone National Park, United States (Chien et al., 1976). Another famous example of thermostable enzymes is DNA polymerase from *Pyrococcus furiosus* (*Pfu* DNA polymerase) (Lundberg et al., 1991). The enormous advances in molecular biology and genetic engineering over the past 4 decades would not have been possible without these extremozymes.

Availability of large numbers of thermophilic extremozymes has opened new possibilities for industry. Thermophilic enzymes have applications in several areas, the most prevalent of which are polysaccharide processing, biofuel production, pulp and paper, and production of fine chemicals and drug intermediates.

#### 2.1.1 Thermophilic Enzymes in Polysaccharide Processing

Enzymatic starch processing involves two steps, liquefaction, which is the dispersion of insoluble starch in an aqueous solution followed by partial hydrolysis with thermostable amylases, and saccharification, the complete degradation of oligomers to monomers with glucoamylases (Li et al., 2015). Liquefaction and saccharification occur at temperatures between 50 and 80°C, necessitating thermostable enzymes. Numerous thermophilic α-amylases, pullulanases, glucoamylases, xylanases and amylopullulanases are commercially available (Sarmiento et al., 2015). Alpha-amylases are typically used in the initial liquefaction step, and glucoamylases and pullulanases are used for subsequent saccharification. Pullulanses and glucoamylases are used in the production of glucose syrups by the food industry, and *ß*-amylases are used for production of maltose syrups by the pharmaceutical industry. Thermostable amylopullulanases are used for production of maltose and maltotriose syrups. Their dual functionality allows simultaneous debranching and liquefaction making them very attractive to the food, beverage and pharmaceutical industries (Li et al., 2021). Submerged and solid-state fermentation processes relying on three thermophilic enzymes, a glucoamylase, amylopullulanase and α-amylase efficiently saccharified starch without the need for calcium or any additional enzymes (Satyanarayana et al., 2004).

#### 2.1.2 Thermophilic Enzymes in Biofuel Production

Biofuels are fuels derived directly from biomass, usually by enzyme-catalyzed fermentations. First-generation biofuels used food crops such as corn, sugar beets and wheat. First-generation biofuels did not succeed as they competed with the global food supply, and will ultimately result in increased food prices (Bhalla et al., 2013). Second-generation biofuels are more applicable, as they utilize lignocellulose, which is often agricultural and forestry waste, is widely available and is inexpensive. Lignocellulose is composed of lignin, cellulose, and hemicellulose. Cellulose and hemicellulose make up approximately two-thirds of lignocellulosic biomass and are the sources for second-generation biofuel production. Lignocellulose has a very rigid, compact structure, and pretreatment prior to enzymatic hydrolysis is necessary so the cellulose/hemicellulose is accessible to enzymes (Alvira et al., 2010). Successful pretreatment strategies require temperatures greater than 50°C, the higher temperature effectively disrupts the structure of lignocellulose and enhances enzyme penetration through the cell wall of the biomass (Bala and Singh 2019). Combination of cellulase and xylanase is the most effective at degradation of lignocellulose because the two enzymes are specific, hydrolysis is nearly complete and fermentation inhibitors are not produced.

Cellulases catalyze depolymerization of cellulose. Cellulase refers to three enzymes, endoglucanase (1,4-β-d-glucan glucohydrolase [EC 3.2.1.4]), *ß*-glucosidase (β-d-glucoside glucohydrolase, [EC3.2.1.21]), and exoglucanase (1,4-β-d-glucan cellobiohydrolase [EC 3.2.1.91]). These three enzymes work synergistically to completely hydrolyze cellulose to glucose (Lynd et al., 2002). Xylanases are needed for hydrolysis of hemicellulose. Xylanase is a group of enzymes comprised of endo-1,4-β-D-xylanases (EC 3.2.1.8), *ß*-D-xylosidases (E.C. 3.2.1.37), ferulic acid esterase (EC 3.1.1.73), acetylxylan esterase (EC 3.1.1.72), α-glucuronidase (EC 3.2.1.139), α-L-arabinofuranosidases (E.C. 3.2.1.55), and p-coumaric esterase (3.1.1.B10). These enzymes act together to degrade the xylan in hemicellulose to monosaccharides and xylo-oligosaccharides (Bhardwaj et al., 2019). Degradation of lignocellulose requires mixtures of several enzymes, and utilizes different schemes including a multienzyme complex called the cellulosome and multifunctional megazymes (Singh et al., 2021).

Multifunctional megazymes are enzymes with at least two different catalytic modules and usually are bifunctional. A hyperthermophilic cellulase/hemicellulase system from *Caldicellulosiruptor bescii* degraded xylan, microcrystalline cellulose, and non-pretreated grass and rice straw at 75–85°C and pH 5–6. The enzyme system contained four multi-domain cellulases, which probably accounted for its high hydrolytic activity. Degradation of rice straw by the *Ca. bescii* enzyme system was 2 fold higher than that of the cellulase/hemicellulose system from the mesophilic fungus *Trichoderma reesei*, which is what is used in commercial enzyme cocktails (Kanafusa-Shinkai et al., 2013). The purified cellulosome from *Clostridium thermocellum* and thermophilic *ß*-glucosidase from *Thermoanaerobacter brockii* reached 91% glucan conversion from pre-treated rice straw, similar to that of commercially available *T. reesei* cellulase Celluclast 1.5 L and Novozyme-188 combination, but with nearly 10 times less enzyme loading, thus presenting an economic advantage (Waeonukul et al., 2012). The cellulosome of *C. thermocellum* achieved 100% glucan conversion of microcrystalline cellulose compared to only 50% conversion by commercial enzyme Cellic CTec2 (Novozyme) (Resch et al., 2013). *C. thermocellum*, when used as a whole cell catalyst, improved carbohydrate solubilization of switchgrass compared to *Ca. bescii* and Novozymes Cellic^®^ Ctec2 and Htec2 (Holwerda et al., 2019). Batch fermentations with non-pretreated switchgrass and *C. clariflavum* resulted in higher solubilization of glucan, xylan and arabinan (64.7–69.4%) in comparison with fermentations with *C. thermocellum* (Izquierdo et al., 2014). All these studies imply that thermophilic enzymes are promising ingredients for cellulolytic cocktails with enhanced biomass degradation and without the need for biomass pretreatment, thereby reducing operational costs and time.

#### 2.1.3 Thermophilic Enzymes in Pulp and Paper Production

The paper industry uses raw lignocellulosic materials (e.g., wood) and wastepaper as raw materials. The first step in paper manufacture is the pulping of wood, which is the removal of lignin associated with the hemicellulosic fraction, preferably without altering the cellulosic fraction. Pulping uses chemical and mechanical processes that involve treatment of lignocellulose with sodium sulfide, sodium hydroxide and caustic soda at 150–170°C, followed by mechanical separation of cellulose from lignin and hemicellulose and bleaching with sodium oxide and chlorine-based chemicals. Traditional pulping processes are not energy efficient and generate large amounts of toxic waste (Gupta et al., 2020). Enzymatic bio-pulping and bio-bleaching overcome many of these problems. Thermophilic/alkaliphilic extremozymes catalyze bio-pulping and bio-bleaching reactions with high efficiency without use of caustic chemicals and often under milder conditions than traditional chemical pulping.

Xylanases are the major enzymes used in bio-pulping, where they hydrolyze xylan present in the hemicellulose of plant cell walls and lignocellulose into xylose monomers. Thermophilic xylanase allows the degradation process to occur concurrently with the initial heating step of lignocellulose with no need for pre-cooling. Xylanases, in combination with lignin-degrading enzymes, are used in bio-bleaching of cellulose pulp as an alternative to chlorine treatment, thereby reducing environmental impact. There are numerous reports on thermostable xylanases. Most commercially available xylanases are of fungal origin and not thermostable (Bhardwaj et al., 2019). The number of thermophilic xylanases commercially available is limited. Verenium corporation supplies two thermophilic xylanases, luminase^®^ PB-100 active between 40 and 70°C and luminase^®^ PB-200 active between 60 and 90°C. Enzyme Deinking Technologies provides EnzAid^®^, active between 50 and 65°C. A recombinant xylanase PRX is available from Sigma Aldrich, active between 45 and 65°C. Megazyme offers 9 fungal and recombinant thermostable xylanases with temperature optima between 50 and 80°C, including recombinant endo-1,4-beta-xylanases from *Thermotoga maritima* and *Geobacillus stearothermophilus* with temperature optima at 80 and 70°C, respectively.

Laccases also play a role in the bio-bleaching of lignin and production of paper pulp. Laccases are oxidoreductases which utilize phenolic compounds as electron donors, and oxygen as an electron acceptor. Lignin is an insoluble complex of phenolic compounds, and thermostable laccases, along with peroxidases, are used in enzyme-based delignification systems, where they separate lignin from cellulose and hemicellulose, and can utilize lignin degradation products as substrates for further reactions (Liu Y. et al., 2020). Laccases reported are produced by thermophiles and hyperthermophiles and have extreme thermostability (Espina et al., 2021). A recombinant laccase from alkalithermophilic *Bacillus* sp. FNT with maximal activity at 70°C is commercially available as an enzyme for the research market from Swissaustral LLC (Athens, GA, United States). Large scale application of thermostable laccases in lignin degradation is very limited however, due to cost of large-scale production, and lack of mechanistic investigation into the enzyme’s stability, activity, and interaction with substrate.

#### 2.1.4 Thermophilic Enzymes in Fine Chemical Synthesis

Transaminases are pyridoxal-5′-phosphate-dependent enzymes which catalyze transfer of an amine group from a donor to a receptor (usually a ketone or aldehyde), resulting in a corresponding ketone/aldehyde and a new chiral amine. The cofactor pyridoxal-5′-phosphate is regenerated at the end of the reaction (Cárdenas-Fernández et al., 2021). Transaminases are used in the synthesis of pharmaceuticals and fine chemicals, particularly chiral amines such as α-, *ß*- and ω-amino acids, synthetic amino acids, and primary amines. An estimated 40% of pharmaceuticals have a chiral amine in their structure (Cárdenas-Fernández et al., 2021). Chiral amine synthesis at high temperature is preferable as it has higher reaction output and lower production cost.

Thermostable transaminases are not currently available commercially. Several thermophilic transaminases were cloned and expressed from thermophiles and hyperthermophiles. Among these, two stereoselective serine-transaminases produced high yields of hydroxypyruvate (70–77%) in the presence of transketolase at 65°C, and retained greater than 80% of maximal activity after 24 h of incubation at 50°C (Cárdenas-Fernández et al., 2021). A number of other thermostable transaminases with maximal activity at 65–80°C have been reported in the literature (Atalah et al., 2019). A moderately thermophilic ω-transaminase successfully synthesized optically pure sitagliptin, a drug for the treatment of type II diabetes, in a process that produced less waste and had higher yield (Desai 2011). The archaeon *Sulfolobus solfataricus* produces a serine transaminase stable at 70°C for 10 min, and was active towards several amino acids other than serine, giving it potential for synthesis of optically pure drug intermediates (Littlechild 2015).

Nitrilases catalyze conversion of nitriles to corresponding carboxylic acids with production of ammonia as a side-product. Nitrilases are used in the synthesis of pharmaceuticals and precursors. A genetically modified, moderately thermophilic nitrilase from *Acidovorax facilis* produced iminodiacetic acid at 40–50°C, a precursor for manufacture of surfactants and chelating agents (Liu et al., 2018). Several other thermophilic nitrilases with maximal activities at 40–65°C have been reported in the literature (Shen et al., 2021), but there are no commercially available nitrilases.

Aminoacylases are of industrial importance because they can resolve racemic mixtures of N-acyl amino acids to L- and d-amino acids and amino acid analogues. Many pharmaceutical compounds contain nitrogen which is derived from amino acids, and they must be optically pure (Littlechild 2015). A thermophilic l-aminoacylase was cloned from the archaeon *Thermococcus litoralis*. It is active at 60–90°C, and is used commercially for production of l-amino acids and analogs by Chirotech/Dow Pharma and Chirotech/Dr Reddys (Littlechild 2015).

### 2.2 Psychrophilic Enzymes

Psychrophilic enzymes have maximal activity between −20 and 10°C. Psychrophilic enzymes are active at low temperature by reducing the energy of activation for reactions, which is achieved by increasing their catalytic rate (k_cat_) and decreasing substrate binding affinity (k_0.5_). This requires reduced enthalpy (less interactions between enzyme and substrate) and increased entropy (changes in structural stability and flexibility) (Santiago et al., 2016). Adaptations in a psychrophilic protein’s structure that allows increased flexibility includes 1) lower arginine/lysine ratio, 2) less proline and more glycine residues in loops, 3) reduced compactness of the hydrophobic core and increased surface hydrophobicity, 4) weaker protein interactions such as fewer hydrogen bonds, disulfide bridges, electrostatic interactions, and weak metal-binding, 5) decreased formation of secondary structure (Sarmiento et al., 2015; Mangiagalli and Lotti 2021). These features preserve enzyme activity at lower temperatures but make the enzyme less stable as the temperature rises beyond 15°C. Psychrophilic enzymes enhance the economics and efficiency of low to moderate temperature processes and enable biocatalysis with lower environmental impact.

#### 2.2.1 Psychrophilic Enzymes in Laundry Detergents and Industrial Cleaning-in-Place

The most advanced market for psychrophilic enzymes is the manufacture of laundry detergents. The global detergent enzymes market is projected to grow with a CAGR of 5.8% over the period 2021–2026 (Research E M 2021). Cold-active laundry detergents reduce energy costs, have a smaller carbon footprint and improve fabric preservation (Kumar et al., 2021). Psychrophilic amylases, proteases, lipases, pectinases, mannanases and cellulases are commercially available (Table 1). Enzymes degrade stains and deposits into soluble products which can then be removed easily by water. Amylases break down starchy stains from fruits, cereals, pasta, potatoes, etc. Proteases hydrolyze proteins in blood, egg, grass, dairy products, sweat, etc, and lipases hydrolyze lipids in grease, oil, butter, sauces. Mannanases and pectinases are active against tough stains due to fruit products, gums, body lotions and other personal care products. Cellulases are active against stains with oats, such as cereals. Cellulases also provide fabric care, they break down accessible broken cotton fibers such as fuzz and pills thus increasing fabric softness and reducing fuzz build-up. In cotton fabrics, cellulase improves brightness by modifying cellulose fibrils (Al-Ghanayem and Joseph 2020).

**TABLE 1:** Commercially available psychrophilic enzymes used in detergents.

Enzyme	Function	Trade name	Source
Protease	Hydrolysis of peptide bonds; removes protein stains	Purafect^®^	Genencor
		Excellase ^®^	
		Purafect Prime^®^	
		Kannase^®^	Novozymes
		Polarzyme^®^	
		Liquanase^®^ Evity ^®^	
		Progress^®^ Excel	
		Progress ^®^ Key	
		Savinase^®^ Evity ^®^	
Mannanase	Hydrolysis of *ß*- 1,4 bonds in mannose polymers, removes guar and locust bean gums	Mannaway^®^	Novozymes
		Mannaway^®^ Evity^®^	
		Effectenz™	DuPont
Amylase	Hydrolysis of α- 1,4 glycosidic bonds in starch, removes starch stains	Amplify^®^ Prime	Novozymes
		Stainzyme^®^ Evity^®^	
		Stainzyme^®^ Plus Evity^®^	
		Preferenz™ S 210	Dupont
		Purafect®OxAm	Genencor
Lipase	Hydrolysis of ester bonds in triacylglycerol, removes fatty, greasy stains	Lipex^®^ Evity^®^	Novozymes
		Lipolase^®^ Ultra	
		Lipoclean^®^	
Cellulase	Hydrolysis of *ß*- 1,4 bonds glycosidic bonds in cellulose, removes damaged microfibrils, soil anti-redeposition, enhance fabric whiteness	Celluclean^®^	Novozymes
		Celluzyme^®^	
		Rocksoft™ Antarctic LTC CONC	Dyadic International Inc
		Retrocell Recop	EpyGen Biotech
		Retrocell ZircoN	
		UTA-88	Hunan Youtell Biochemical
		UTA-90	
Pectate lyase	Hydrolysis of α- 1,4 linked polygalactosyluronic acid, removes fruit and pectin-based stains	XPect^®^	Novozymes

Psychrophilic enzymes are also used for cleaning-in-place by the food, brewing and dairy industries. Enzymes clean equipment and deblock filters used in the beverage industry. Lipases, proteases, amylases and pullulanases degrade molds and biofilms in filters and building surfaces, thus allowing for greater cleaning performance without the use of chemical detergents, surfactants or organic solvents (Barroca et al., 2017).

#### 2.2.2 Psychrophilic Enzymes in the Pharmaceutical Industry

Psychrophilic enzymes have been applied commercially in the pharmaceutical industry. A cold-active lipase from *Candida antarctica* was described with activity against long- and medium chain primary alcohols and carboxylic acids as well as secondary and tertiary alcohols, giving it potential for application in organic syntheses of cosmetic, pharmaceutical and flavor compounds (Kirk and Christensen 2002). CALB (*Candida antarctica* lipase B) has a wide range of applications, including polysaccharide modification and resolution of alcohols and amines during synthesis of calcium antagonists, cosmetics and fragrance esters (Huston 2008). The immobilized form of CALB, Novozym 435, is widely used commercially and has been applied in esterification reactions for production of fine chemicals, production of optically pure products and in amidation reactions (Ortiz et al., 2019). Other psychrophilic enzymes have been shown to produce flavor esters by esterification at 10–20°C (Mhetras et al., 2021).

Psychrophilic proteases have diverse therapeutic applications, due to more effective catalysis of biological processes at lower temperatures compared to mesophilic proteases and good safety profiles. Psychrophilic proteases have good efficacy in topical applications; the enzymes greater flexibility allows high activity in low water conditions encountered around membrane proteins and within the lipid layer of mucus. They also have a localized effect, the psychrophilic protease will have high activity when applied externally at ambient temperature, then will lose activity when reaching equilibrium with the warm, *in vivo* environment (Fornbacke and Clarsund 2013). Psychrophilic proteases from Antarctic krill (acidic endo- and exopeptidases) were superior to a saline control in debridement of necrotic ulcers in a model of wound recovery. They improved tissue granulation, reduced degree of necrotic tissue and enhanced wound healing with negligible systemic side effects (Mekkes et al., 1998).

#### 2.2.3 Psychrophilic Enzymes in Molecular Biology

Cold-adapted alkaline phosphatases, nucleases, uracil-DNA N-glycosylases, proteases and DNA ligases are commercialized as molecular biology tools by several companies (New England Biolabs, ArcticZymes, Takara-Clontech, Affymetrix) (Barroca et al., 2017; Mangiagalli et al., 2020). Psychrophilic enzymes are unstable at temperatures greater than 20°C and can be inactivated by moderate heat treatment, making them very attractive in molecular biology applications. This precludes the need for time-consuming chemical, or column-based purification procedures, and moderate heat treatment preserves the integrity of RNA and double stranded DNA for subsequent processes.

Alkaline phosphatase dephosphorylates the 5′end of linear DNA to prevent re-circularization during cloning. Antarctic phosphatase, originally isolated from an Antarctic bacterium and improved by directed evolution is available commercially by New England Biolabs (Ipswich, MA, United States). ArcticZymes (Tromsø, Norway) provides a cold-adapted alkaline phosphatase from arctic shrimp (Sarmiento et al., 2015). Double stranded nuclease digests double stranded DNA with no effect on single stranded DNA, such as probes. Double stranded nuclease is used to remove DNA from RNA preparations (Mangiagalli et al., 2020). Heat labile nucleases are available from Affymetrix and ArcticZymes, they are inactivated after exposure to 65–70°C for 30 min. Uracil-DNA N-glycosylase removes uracil from uracil-containing DNA. They are used in PCR, reverse-transcriptase PCR and site-directed mutagenesis single nucleotide polymorphism genotyping. Several cold-active uracil-DNA N-glycosylases are available commercially with maximal activity at 20–25°C and inactivation temperatures 40–50°C (Sarmiento et al., 2015; Mangiagalli et al., 2020).

Proteases remove protein contamination from DNA and RNA preparations. Psychrophilic proteases are advantageous, their heat-lability allows inactivation at temperatures that do not compromise structure of RNA and DNA. ArcticZymes produces a cold-stable proteinase of marine origin (Mangiagalli et al., 2020). DNA ligase catalyzes formation of 3′- 5′-phosphodiester bonds between deoxynucleotides to join DNA fragments with staggered or blunt ends. Annealing of staggered DNA ends is more efficient at temperatures between 3 and 10°C. Currently available ligases from T4 and T7 bacteriophages have maximal activity at 37°C and are very slow at lower temperatures, forcing ligation times exceeding 12 h. To this end, a psychrophilic ligase will be beneficial as it will provide efficient ligation at the low temperature required for annealing. Reports on psychrophilic DNA ligases are scarce, and none are commercially available. Vib-Lig, a psychrophilic DNA ligase, was cloned from the obligate psychrophile *Aliivibrio salmonicida*. It has maximal activity at 20°C and was active over the temperature range 10–35°C (Berg et al., 2019). A psychrophilic DNA ligase from Antarctic Sea water bacterium *Pseudoalteromonas haloplankis* was cloned, and had maximal catalytic efficiency at 18°C, and retained greater than 50% of maximal efficiency at 4°C (Georlette et al., 2000).

#### 2.2.4 Psychrophilic Enzymes in the Food and Beverage Industry

Enzymes have been used as food ingredients, food additives and processing aids for hundreds of years. Enzymes are used in traditional processes of brewing, bread making and cheese manufacturing, and in recent applications such as production of nutraceuticals and functional foods. The trend over the past decade has been to replace high-temperature processes with low-temperature processes. Low-temperature processes have smaller environmental impact, lower cost, reduced microbial/fungal contamination and spoilage and enhanced shelf-life (Barroca et al., 2017).

Nearly 70% of the global population is unable to digest lactose after infancy. Β-galactosidases degrade lactose to glucose and galactose and can be used to remove lactose from dairy products to enhance digestibility. Psychrophilic *ß*-galactosidases are advantageous as they can efficiently hydrolyze lactose in dairy products at storage temperature (4–8°C) in a batch process before pasteurization and packaging (Mangiagalli and Lotti 2021). Psychrophilic *ß*-galactosidases can also be used in valorization of whey, a side-product of cheese production. Hydrolysis of whey by *ß*-galactosidase produces glucose- and galactose-rich syrups that can be used as sweeteners (Kumari et al., 2021). *ß*-galactosidases also have transglycosylation activity, where hydrolysis of lactose is accompanied by formation of tri- and tetra saccharides, which, in addition to being low calorie sweeteners, have potential use as prebiotics as they enhance growth of *Bifidobacteria* in the intestine (Benešová et al., 2005). Production of oligosaccharides has also been reported by psychrophilic *ß*-galactosidases from marine bacteria *Alteromonas* sp. ANT48, *Marinomonas* sp. BSi20414, *Pseudoalteromonas* sp. 22b and *Arthrobacter* sp. 32bc (Mangiagalli and Lotti 2021). Most of the psychrophilic *ß*-galactosidases reported originate from the Antarctic and Arctic, and are active over a broad temperature range, from 4–50°C (Kumari et al., 2021). The archaeon *Halorubrum lacusprofundi* produces a *ß*-galactosidase active from −5–60°C (Karan et al., 2013). The first step in the production of the low-calorie sweetener tagatose uses a psychrophilic *ß*-galactosidase (Van de Voorde et al., 2014). The dairy industry also makes use of cold-adapted proteases, lipases and phospholipases during cheese production and ripening (Barroca et al., 2017; Mangiagalli et al., 2020).

Enzymes used in baking applications improve product quality, reduce polyacrylamide production, and minimize use of chemical additives. Most enzymes currently applied are mesophilic and thermotolerant but preparing dough at moderate temperatures offers a significant cost advantage. Xylanases have an essential role in bread-making, they degrade hemicellulose to soluble sugars resulting in soft and elastic bread. There are limited reports on psychrophilic xylanases. Three psychrophilic xylanases from *Flavobacterium* sp. MSY-2, *Pseudoalteromonase haloplanktis* and an unidentified bacterial isolate improved dough flexibility and crumb structure compared to commercially available, mesophilic xylanases (Dornez et al., 2011).

Psychrophilic proteases, lipases and chitinases have been applied in fish descaling, skin removal and degreasing and oil extraction (Barroca et al., 2017). Psychrophilic proteases are used for meat tenderization and flavor enhancement. A psychrophilic protease from *Pseudoalteromonas* sp. SM9913 released more taste amino acids from the surface of marine fish, shrimp and pork than a mesophilic protease at 0°C (He et al., 2004).

Pectinases are a group of enzymes comprising pectin esterases, pectate lyases and polygalactoronases, which degrade pectin. Pectin is a fiber found in plants and fruits. Pectinases are used for fruit and vegetable processing, where they improve clarification, increase extraction yield, and reduce viscosity of fruit juice. A very limited number of psychrophilic pectinases are commercially available. Lallzyme^®^ is a mixture of pectin lyase, pectin esterase and polygalacturonase from *Asperigillus niger*, active between 5 and 20°C, and is used or clarification of juices and wine at ambient temperature (Sarmiento et al., 2015).

### 2.3 Acidophilic Enzymes

Acidophilic enzymes have maximal activity at pH values less than 3. The structural adaptations needed to retain stability at low pH have not been studied in detail, but reports on acid-stable amylases showed an abundance of acidic amino acids (aspartate, glutamate) and less basic amino acids on the surface of the proteins (Parashar and Satyanarayana 2018). Acidophilic amylases have acidic isoelectric points, ranging between 4 and 5. At pH values less than 3, most of the surface amino acids will be protonated resulting in a less negative charge and protein stabilization. As the pH increases, more amino acids will become deprotonated, and unfolding will occur due to repulsion between the excess negative groups. Acid stable enzymes have numerous applications, particularly in the production of sugar syrups from starch, bioremediation and in microbial fuel cells.

#### 2.3.1 Acidophilic Enzymes in Starch Processing

Production of oligosaccharide syrups from starch involves three steps: gelatinization of starch slurry at 90–100°C, liquefaction with α-amylase, and digestion with glucoamylases. The pH of the native starch slurry is between 3 and 5. Acidophilic amylases will negate the need to neutralize the starch slurry before liquefaction, thus reducing the time and cost of oligosaccharide production from raw starch. There are several reports on acidophilic amylases and glucoamylases. The majority are produced by acidothermophilic bacteria (*Bacillus, Alicyclobacillus*) and archaea (*Thermoplasma, Sulfolobus, Picrophilus*), and have maximal activities at pHs between 3 and 6 (Bertoldo et al., 2004; Sharma et al., 2016; Parashar and Satyanarayana 2018). Commercially available acidophilic amylases are limited however, mainly due to little secretion of extracellular amylases by acidophilic microorganisms. Large scale heterologous expression of acidophilic amylases has also been unsuccessful due to poor expression and formation of inclusion bodies (Parashar and Satyanarayana 2018). Genencor International (Palo, Alto, CA, United States) produces two acidophilic amylases: Stargen™ 001 active at pH 4.0–4.5, and Spezyme Xtra, active at pH 5.5–6.0. Valley Research (South Bend, IN, United States) produces Ultra-Thin, active at pH 4.5. Novozymes (Franklinton, NC, United States) produces several amylases: Liquozyme^®^ SC, active at pH 5.7–6.0, Liquozyme^®^ Supra 2.2X active at pH 5.5, LpHera^®^ active at pH 4.5, and Termamyl^®^ SC DS, active at pH 5. Novozymes also produces Spirizyme ^®^, a mixture of glucoamylases active at pH 3.5–5.5.

#### 2.3.2 Acidophiles in Bioremediation

Acid mine drainage (AMD) forms when sulfide minerals in the Earth are exposed by mining or major construction projects. Most sulfide minerals oxidize to sulfuric acid when exposed to water and oxygen, which then enter the surface and groundwater. AMD is a major reason for water pollution and dispersion of heavy metals into the environment. AMD has a pH between 2 and 8 and is filled with metals and sulfides. Conventional methods for treatment of AMD involved alkalinization of the acidic effluent beyond the optimal pH requirements of iron-oxidizing bacteria and/or treating the acidic effluent with crushed limestone to reduce the rate of acid generation (Skousen et al., 2019). These methods are not efficient, have high operational costs, and generate large quantities of solid sludge that needs to be disposed of.

AMD is inhabited by acidophilic microorganisms, mainly *Acidiphilum, Acidithiobacillus, Acidisphaera*, and *Leptospirillum* (Li et al., 2020). In addition to being acidophilic, these microorganisms are capable of oxidation and reduction of iron and sulfur, and are resistant to toxic metals such as cadmium, chromium, nickel, and arsenic. AMD bioremediation strategies do not use individual enzymes but whole cells, and consist of bioreactors containing acidophilic iron-oxidizing bacteria (e.g. *Leptospirillum ferroxidans*) and sulfate-reducing bacteria (e.g. *Acidithiobacillus ferroxidans, A. ferrivorans*). These microorganisms secrete extracellular oxidoreductases that are stable at pH values much lower than their cytoplasmic pH (∼ pH 5) (Sharma et al., 2012). AMD flows through the bioreactors, and fermentation creates conditions that allow sulfate reduction and metal precipitation (Villegas-Plazas et al., 2019). Sulfate reduction produces alkalinity by converting sulfate to sulfide. Dissolved metals then bind to sulfide forming insoluble metal sulfides. Neutralized, metal-free drainage then leaves the bioreactor. Several examples of AMD treatment bioreactors which have reached >90% heavy metal removal have been described (Johnson and Hallberg 2005; Chen et al., 2016; Rodrigues et al., 2019; Sun et al., 2020).

#### 2.3.3 Acidophiles in Microbial Fuel Cells

Microbial fuel cells (MFCs) are a promising technology that allow concomitant generation of bioelectricity and bioremediation of wastewater. An MFC consists of two compartments (anode and cathode) separated by a proton-selective membrane. In the anodic compartment, electrochemically-active microorganisms oxidize organic and/or inorganic material in waste, releasing electrons, protons and carbon dioxide. The electrons move towards the cathode via an external electrical circuit, where oxygen is usually the electron acceptor. The kinetics of oxygen reduction are slow and associated with large overpotentials. Use of acidophilic iron-oxidizing bacteria, such as *A. ferroxidans* at the cathode compartment greatly improved the kinetics of oxygen reduction at acid pH (Izadi et al., 2019). Even though *A. ferroxidans* is capable of iron oxidation at neutral pH, reduction of oxygen and energy gain is greater at pH 2 (Ferguson and Ingledew 2008). An MFC using a mixed culture of *AAcidithiobacillus* sp, *Ferroplasma* sp. and *Leptospirillum* sp. as cathode biocatalysts had a four-fold increase in electrical power output (Stom et al., 2021). Arsenic and iron were successfully removed from AMD by a single chamber air cathode MFC using microorganisms from acidic iron/arsenic rich surface sediments by surface sorption and bioprecipitation (Leiva et al., 2018).

Presence of acidophilic microorganisms in the anodic compartment is also advantageous. The internal resistance of an MFC decreases as the pH difference between the anode and cathode compartments increases, leading to greater power output (Jadhav and Ghangrekar 2009). A single chamber microbial fuel cell operating using anaerobic anodic biocatalysts had greater power output when the anodic pH was maintained at 6, compared to anodic pH 7 or 8 (Sharma et al., 2016). Unfortunately, large scale use of MFCs remain limited due to low power outputs and cost associated with scale up.

### 2.4 Alkaliphilic Enzymes

Alkaliphilic enzymes have maximal activity at pH values greater than 9. They have more alkaline isoelectric points relative to their neutrophilic counterparts, due to abundance of arginine and histidine residues, and decrease in glutamate residues. Arginine residues form ionic bonds with aspartate residues, which is hypothesized to be critical for enzyme stability in alkaline conditions (Fujinami and Fujisawa 2010). The first large scale application of alkaliphilic enzymes was as additives to laundry detergents. Alkaline lipases, proteases, amylases and cellulases are stable in alkaline detergents commonly used in laundry machines and dishwashers, resist denaturation effect of surfactants and effect of chelators such as ethylene diamine tetra-acetic acid, and are generally stable during long term storage and in the presence of bleaching agents (Fujinami and Fujisawa 2010).

Alkaliphilic proteases have also been applied in leather processing. Leather consists mainly of collagen and is associated with other globular and fibrous proteins such as globulin, albumin, keratin and elastin. During leather processing, hair attached to the skin is removed, as well as non-collagenous material such as fat and flesh. Conventional leather processing uses large amounts of lime, acid, sodium sulfide, soap, dyes, solvents and thereby producing toxic waste (Wanyonyi and Mulaa 2020). Enzymatic leather processing reduces time, environmental impact, and cost of leather processing. Soaking of hides and skins in alkaline solutions with proteases successfully removes non-collagen protein and hair and initiates fiber opening of the skin and hide. The alkaline pH greatly reduces microbial contamination which is a major problem in conventional soaking. Enzymatic dehairing of hides and fish skin with alkaline protease completely replaced lime and sodium sulfide (Wanyonyi et al., 2016). Alkaline lipases are used to remove natural fat from skins and hides in tanneries, where alkaline lipases improve color and appearance of the finished product and produce waterproof leather (Hasan et al., 2006).

### 2.5 Halophilic Enzymes

Halophilic enzymes have maximal activity at sodium concentrations greater than 1.5 M. The source of sodium is mainly sodium chloride but can also be sodium carbonate/bicarbonate. Many halophilic enzymes can also tolerate molar concentrations of potassium. Enzyme adaptations to high salinity include having an acidic isoelectric point and an abundance of aspartate and glutamate residues on the enzyme surface (Oren 2013). Acidic amino acids have superior water-binding abilities which keep the protein soluble under dehydrating conditions caused by salinity. Many halophilic enzymes tolerate organic solvents because organic solvents reduce water activity like high salinity does. Another adaptive mechanism is a decrease in solvent-exposed hydrophobic amino acid residues and a smaller protein core. Weak hydrophobic interactions cause increased protein flexibility at high salt concentrations because a weak hydrophobic core will not become too rigid in an ionic environment (Brininger et al., 2018).

Halophilic enzymes have great potential for application in biotechnology, bioremediation, and biopharmaceutics; they retain stability and solvation at low water conditions and can be scaled up in processes with organic solvents and brine without strict sterile conditions. However, large scale production and application of halophilic enzymes remains under-developed compared with other extremozymes. This is due to mechanical fragility of many halophilic microorganisms, particularly haloarchaea which are prone to lysis in low salinity, and large scale culturing of halophilic microorganisms can lead to damage and corrosion of stainless steel equipment (Haque et al., 2020). Large scale heterologous expression of halophilic proteins can also be problematic, as many halophilic proteins misfold and aggregate at low salinity (Martínez-Espinosa 2020).

Halophilic proteases and amylases have been included in detergent formulations (Patel and Saraf 2015; Mokashe et al., 2018). NaCl is an ingredient used in manufacture of granular detergents, thus salt stability of enzymes is a must. Halophilic extremozymes are used in biocatalysis in non-conventional and organic solvents, either in free or immobilized form. Halophilic cellulases, xylanases and laccases can be used in the production of biofuels and have application in processes where lignocellulose is pretreated with organic solvents or ionic liquids (Mesbah and Wiegel 2017; Amoozegar et al., 2019; Kasirajan and Maupin-Furlow 2021; Motamedi et al., 2021). Free and immobilized lipases from *Haloarcula* sp. G41 and *Bacillus lichenformis* produced biodiesel with yields greater than 85% from soybean oil and Myrtus oil (Qiu et al., 2021). Halophilic lipases and esterases have applications in the synthesis of short chain and poly-unsaturated fatty acids, which are used in nutraceuticals and dietary supplements (Salihu and Alam 2015). A lipase from halothermophile *Rhodothermus marinus* synthesized the aroma ester methyl acetate in n-hexane in free and immobilized form (Memarpoor-Yazdi et al., 2017). The chemo- and regioselectivity of halophilic lipases allows them to be used in production of lipophilic antioxidants by modification of natural polyphenols (Delgado-García et al., 2018).

Halophilic extremozymes have also been incorporated in reverse micelles and can be used for development of biosynthetic processes in non-aqueous media. Archaeal halophilic enzymes p-nitrophenylphosphate phosphatase (pNPPase) and malate dehydrogenase (hMDH) were successfully encapsulated in reverse micelles with cetyltrimethylammonium bromide in cyclohexane (Marhuenda-Egea and Bonete 2002). Many halophilic enzymes, particularly the archaeal enzymes, unfold and lose activity at low salt concentrations. Maintenance of catalytic activity of halophilic enzymes in reverse micelles removes a major obstacle to their large scale application (Singh and Singh 2017).

### 2.6 Radiophilic and Barophilic Enzymes

Radiophiles survive and grow in environments with high oxidative stress and radiation, including gamma radiation, ultra-violet radiation, and X-ray radiation (Sysoev et al., 2021). There are no reports on isolation of radiophilic enzymes; radiophilic microorganisms have been used as whole cells in decontamination of radioactive waste environments and bioprecipitation of uranium from diluted nuclear waste (Brim et al., 2003; Appukuttan et al., 2006). Extremolytes produced by radiophiles have potential applications. Radiophiles can repair extensive DNA damage, and their extremolytes can be used in prevention and treatment of cancer. Mycosporine-like amino acids from the red alga *Porphyra rosengurttii*, when applied topically, prevented sun burn cell formation and other structural alterations in skin of hairless mice, (De La Coba et al., 2009). Deinoxanthin produced by the radiophile *Deinococcus radiodurans* induced apoptosis in three human cancer cell lines, giving potential as application as an anti-cancer agent (Choi et al., 2014). Bacterioruberin produced by radiophiles *Halobacterium* and *Rubrobacter* can prevent skin cancer as it can repair DNA damage caused by ionizing radiation (Raddadi et al., 2015).

Barophiles are microorganisms that tolerate high pressure, with optimal growth at 70–80 MPa. Barophiles have been isolated from the deep ocean. Reports on barophiles are scarce, proteins produced by barophiles have been used in sterilization of food items at high pressure (Arora and Panosyan 2019).

## 3 Bioprospecting of Extremozymes From Extreme Environments

### 3.1 Bioprospecting and the Challenges of Cultivation

Bioprospecting is the discovery and commercialization of novel products from biological sources. Traditionally, bioprospecting of extremozymes has been done by culture-dependent approaches, where culture media and conditions are designed to enrich for microorganisms with properties and activities of interest. Culture-dependent approaches have led to discovery of numerous extremozymes, especially hydrolases (amylase, protease, cellulase, lipase) (Ferrer et al., 2007). The increased prevalence of whole genome sequencing allowed for sequencing of extremophilic genomes and cloning and characterization of putative extremozymes (Elleuche et al., 2014; Suriya et al., 2016; Gallo et al., 2021).

However, less than 1% of environmental microorganisms are culturable in laboratory settings (Amann et al., 1995). There are many reasons for uncultivability of microorganisms. These include lack of specific nutrients or cofactors (produced by other members of the environment or present within the environment), inadequate pH, osmotic pressure, light or oxygen (Vester et al., 2015). Mimicking extreme environments in the laboratory for cultivation of extremophiles is labor intensive and expensive as it requires specific equipment such as high/low temperature incubators, high pressure incubation systems, UV incubators, and culture vessels resistant to corrosion from high acidity/alkalinity/salinity. Lack of sufficient knowledge on media components and long incubation times further complicate culturing. Innovation culture technologies such as cultivation chips, microfluidics, single-cell manipulation and high-throughput cultivation (culturomics) have been developed for mesophilic microorganisms and are difficult to apply in cultivation of extremophiles (Gutleben et al., 2018). The challenges posed by cultivation of extremophiles make culture independent approaches essential to be able to tap into the vast biotechnological potential of extremophiles and their enzymes.

Environmental genomics involves bioinformatic and/or functional analysis of bulk DNA from an environment and provides access to all members of the community including aggregated cells and cells adhered to solid surfaces (Hedlund et al., 2014). Advances in culture-independent omics approaches, such as metagenomics, function-based genomics, and single cell genomics, has provided insight into phylogeny and metabolic capabilities of the uncultivated majority. Genomics based studies focusing on extreme environments have revealed putative halophiles (Nanohaloarchaeota) (Narasingarao et al., 2012), thermophiles (Acetothermia, Atribacteria, Koracheota), acidophiles (Parvarcheota) and piezophiles (Gracilibacteria) (Hedlund et al., 2014). Genomics data provides insights into the physiology and biology of these microorganisms, including anabolic and catabolic potential. However, the major limitation of environmental genomics lies in the fact that annotation algorithms currently available rely on existing functional annotations, and do not provide reliable annotation of novel and under-studied taxa (Grötzinger et al., 2018; Attrill et al., 2019). Furthermore, structural and functional characteristics of enzymes can be altered when in recombinant form. Culture-dependent methods cannot be rendered obsolete, as they are still needed for study of novel enzymes structure and function (Speda et al., 2017).

### 3.2 Metagenome Based Bioprospecting

Metagenomics involves isolating DNA directly from the environment. Analysis can either be sequence-based, where putative enzymes are identified based on conserved regions, or function-based, where genes are cloned, expressed, and screened for activity.

#### 3.2.1 Sequence-Based Metagenomics

Sequence-based metagenomics used to rely on colony hybridization for screening of metagenomic clones using DNA probes for the target gene. Desired genes could then be amplified by PCR using degenerate primers and then cloned into expression vectors. The advent of high throughput sequencing circumvented the need for cloning before sequencing, which was a major limitation of large-scale sequencing. Following bioinformatic annotation, promising sequences identified from metagenomics data can be synthesized *de novo*, codon-optimized, cloned and heterologously expressed. In theory, this method allows for large scale production of extremozymes and/or extremolytes without the need for isolation and cultivation of the source extremophile.

Despite the increased availability and decreasing cost of high throughput sequencing, and enhanced access to extreme environments for sampling, sequence-based metagenomics has not led to a burst in availability of novel extremozymes. The major limitation is availability of robust bioinformatic analyses. Gene annotation tools are homology-based and depend on functional groups that have already been described. Thus, novel and/or non-homologous activities can be missed, and sequences from novel taxa cannot be identified. Large data size and short DNA reads pose analytical challenges. Assembly of genomes of microorganisms that are not abundant in the environment is difficult (Sysoev et al., 2021).

Nevertheless, there are reports on successful production of extremozymes from sequence-based metagenomes. Many of them are thermophilic hydrolases (Sysoev et al., 2021). A halophilic, thermostable mercuric reductase was characterized from the metagenome of the deep brine environment of the Red Sea. This mercuric reductase had maximal activity at 4 M NaCl and 40–50°C and efficiently detoxified Hg^2+^ (Sayed et al., 2014). An alkali tolerant, mildly thermostable laccase was identified from a marine microbial genome of the South China Sea, cloned and heterologously expressed. The enzyme, named Lac15, was active over pH range 6.5–9.0 and 15–45°C, and was able to decolorize dyes under alkaline conditions (Fang et al., 2011). An acidophilic polyphenol oxidase was characterized from metagenomic libraires from bovine rumen microbiota (Pérez-Llano et al., 2020). Two thermophilic esterases, EstE1 and Est 1 were isolated from metagenomic libraires constructed from thermal areas in Indonesia and Thailand. Both enzymes had maximal activity at > 70°C, and retained activity at 30°C (Lopez-Lopez et al., 2014). A psychrophilic lipase LipCE was identified in metagenome of oil-contaminated soil in northern Germany, with activity at 0–30°C (Elend et al., 2007).

Thermostable amine transferases, carbonic anhydrases, and *ß*-xylosidases were isolated from hot spring and hydrothermal vent metagenomes (Yadav et al., 2019). Three novel (S)- selective amine transaminases were discovered in metagenomes constructed from hotsprings in Italy and Iceland, cloned and functionally characterized. One of the transaminases, B3-TA, was extremely thermophilic, with maximal activity at 90°C and retaining over 80% of maximal activity after incubation for 5 days at 80°C. The transaminases catalysed amine transfer reactions with a range of donor and acceptor substrates, making them promising candidates for large-scale production of optically pure amines (Ferrandi et al., 2017). Two thermostable epoxide hydrolases were isolated from metagenomes constructed from hot springs in Russia and China, cloned and expressed. The enzymes had maximal activity at 45–60°C, and hydrolysed a broad range of epoxides, giving them potential for production of optically pure epoxides and diols, which are precursors for preparation of fine chemicals and drugs such as *ß*-blockers (Ferrandi et al., 2015). The development of more flexible metagenomics analysis software and annotation tools is expected to lead to identification and large-scale production of more extremozymes.

#### 3.2.2 Function-Based Metagenomics

Function-based metagenomics involves cloning random fragments of environmental DNA into expression hosts to form a library, then screening the library for different enzyme activities. The advantage of this method is that it identifies active enzymes and does not rely on homology-based gene annotation. However, several factors must be optimized for the technique to be successful, including DNA fragment size, expression vectors, expression host, choice of activity assay, assay conditions, and assay time. As is the case for many extreme environments, the concentration of DNA extracted may not be sufficient for library construction and must undergo whole genome amplification (Taupp et al., 2011). Gene expression may fail due to weak promoter recognition, low translation efficiency and protein misfolding. Expression of archaeal enzymes may fail due to absence of necessary post-translational modifications in bacterial hosts (Eichler 2020). Function-based metagenomics is also biased towards enzymes that function under standard laboratory conditions. Extreme conditions required by many extremozymes for activity, such as high pressure, high salinity, acid/alkaline pH, high/low temperature may be difficult to implement and can inhibit growth of expression hosts.

Several extremozymes have been discovered by function-based metagenomics (Vester et al., 2015; Yadav et al., 2019; Datta et al., 2020; Sysoev et al., 2021). The majority of extremozymes discovered are hydrolases, particularly esterases and lipases. This is most likely due to ease of detection of these activities. Most of the function-based screenings used *E. coli* as an expression host. Development of alternative expression hosts (archaeal in particular) and assays are needed to enable detection of additional extremozyme activities.

### 3.3 Single Amplified Genomics

Single cell genomics involves separation of individual cells from a mixture, followed by cell lysis, amplification of genomic DNA and genome construction. There are plenty of methods for isolation of single cells, including fluorescence-activated cell sorting (FACS) (Sysoev et al., 2021), serial dilution, optofluidics (optical trapping of cells in a microfluidic device (Landry et al., 2018), and micromanipulation (Woyke et al., 2010). FACS is the most used method due to ability to separate cells based on morphology and size. The disadvantage of all cell sorting methods is low efficiency in separating cells that are not abundant in the population and inability to identify cells (Sysoev et al., 2021).

Cells must be lysed after sorting, which is done via standard techniques (e.g. heat, alkaline solution, detergent). The very small amount of DNA obtained must then be amplified to allow sequencing and contig formation. Genome amplification is typically done by multiple displacement DNA amplification. The resultant single amplified genome is then sequenced, assembled, and analysed for genes of interest. Single amplified genomics has many challenges. The large amount of genome amplification required increases the probability of contamination and introduces chimeras which result in bias in genomic coverage (Hedlund et al., 2014). Numerous methods have been developed to address problems of single amplified genomics data, chimeras can be overcome by combining sequence data from several, closely related cells (nucleotide identity >95%) (Rinke et al., 2013). A jackknifing procedure was developed to remove chimeric sequences from multiple single-cell genome sets (Dodsworth et al., 2013).

### 3.4 Challenges of Gene Function Annotation

Whether sequence-based, function-based, or single amplified genomics, all face the challenge of gene function annotation. Most available sequence-based data on enzymes is from mesophiles. Extremozymes have altered amino acid composition to adapt to harsh conditions, and this affects homology-based searches. The number of characterized extremozymes is very limited, and thus sequence data for extremozymes is under-represented in databases. Annotation reliability is inversely related to protein diversity, thus correct annotation of extremozymes is difficult. False negative hits due to incomplete annotations will miss many potential extremozyme sequences. On the other hand, false positive hits from incorrect annotation have very time consuming and expensive consequences, as experimental characterization of enzymes from single amplified genomes involves gene synthesis, amplification, cloning, expression, purification, and activity assays. This has led to development of algorithms to minimize false positive annotations of enzymes. A Profile and Pattern Matching (PPM) strategy was developed the utilizes InterPro gene ontology terms and relevant annotated PROSITE IDs, and allows identification of protein sequences from databases with limited annotation reliability, such as when single amplified genomes of extremophiles are used (Grötzinger et al., 2014). Using the PPM algorithm, a halophilic γ-carbonic anhydrase, glucose dehydrogenase, protease and 2-hydroxy dehydrogenase were identified from single amplified genomes of uncultured archaea from the deep brine pools of the Red Sea, expressed and characterized by X-ray crystallography (Vogler et al., 2020; Sysoev et al., 2021). An alcohol dehydrogenase from an uncultured halo-thermophilic archaeon from a Red Sea brine pool was also expressed and characterized by crystallography (Grötzinger et al., 2018).

## 4 Conclusion

The biodiversity of nature provides an ever-increasing source of organisms which can be exploited for a wide range of biotransformations with commercial interest. Extremozymes are robust biocatalytic tools and catalyze various degradation, synthesis, redox and transformation reactions with high specificity and selectivity under harsh industrial conditions. The numerous limitations on culturing of extremophiles for extremozyme production necessitates development of novel molecular technologies. Successful bioprospecting of biocatalysts of extreme environments is mediated by culture-independent technologies, such as metagenomics, function-based genomics, and single amplified genomics. The tremendous progress in DNA and RNA sequence analysis made over the past 2 decades, combined with development of algorithms and bioinformatic tools tailored to analysis of genomic data from extremophiles, is an enormous step forward in exploration of unknown bacterial and archaeal communities of extreme environments, and will further detection and application of extremozymes in industrial processes thereby paving the way for a bio-sustainable future.
